# Editorial: Atrial fibrillation in dementia

**DOI:** 10.3389/fcvm.2023.1244294

**Published:** 2023-07-07

**Authors:** Michael Gotzmann, Matteo Anselmino

**Affiliations:** ^1^ Department of Cardiology and Rhythmology, St. Josef-Hospital, Ruhr University Bochum, Bochum, Germany; ^2^ Division of Cardiology, Department of Medical Sciences, “Città Della Salute e Della Scienza di Torino” Hospital, University of Turin, Turin, Italy

**Keywords:** atrial fibrallation, atrial flutter, dementia, dementia after stroke, dementia alzheimer

**Editorial on the Research Topic** Atrial fibrillation in dementia

Atrial fibrillation (AF) and dementia are diseases with a significantly increasing incidence worldwide, associated with increased morbidity and mortality and high costs for national health systems. One the one hand, the lifetime risk of developing AF affects one in three persons. On the other hand, ischemic strokes, a leading contributor to dementia, are caused by AF in 20%–30% (1).

Several pathological mechanisms are suspected to be responsible for the association between AF and cognitive decline (2). As AF has a strong association with stroke, there is an association between AF and vascular dementia (3–7).

In this special issue of Frontiers in Cardiovascular Medicine, Gao et al. performed a Mendelian randomization analysis to investigate the causal genetic association between AF and vascular dementia. The authors were unable to provide evidence for a causal relationship between atrial fibrillation and vascular dementia; nevertheless, this finding is important for understanding the pathophysiology of the two diseases (Gao et al.).

Several longitudinal studies in the past provided evidence that an association between AF and dementia may exist even without prior stroke (8). A meta-analysis included studies with more than 75,000 patients without a history of stroke and normal baseline cognitive function. In this meta-analysis, a total of 15% of all patients had AF. After a median observation period of approximately eight years, 6.5% developed dementia. Baseline AF was independently associated with the later occurrence of dementia (5). In addition, the effects of AF on cognitive function were evaluated by the Cardiovascular Health Study which included over 5,000 participants without prior stroke. AF occurred in 11% of patients and was associated with more rapid deterioration of mean cognitive function compared with patients in sinus rhythm (6).

In this special issue, Chen et al. highlight that AF is associated with a significantly worse prognosis even in patients who already have dementia. However, the authors suggest that oral anticoagulation and antiarrhythmic medication of AF is associated with a better outcome. The finding should motivate geriatricians and cardiologists to optimally treat AF also in patients with pre-existing dementia (Chen et al.).

In this special issue, Wang et al. address the question of whether atrial flutter carries a similar risk of dementia compared to AF. The question is of interest because atrial flutter carries a similar (but not identical) risk of stroke compared to AF. In this large epidemiological study, data from Taiwan’s National Health Insurance Research Database were analyzed over a 12-year period. Dementia occurred in 9.82% of patients with AF and in 6.88% of patients with atrial flutter (*p* < 0.001), a finding that remained significantly different even after propensity score matching (Wang et al. 9). The result could indicate that the deleterious effect of AF on cerebral function, irregular blood pressure amplitudes in AF may be an independent factor in the development of cognitive dysfunction. It has been described previously that altered cerebral perfusion (10) and hippocampal atrophy in patients with AF may be due to irregular R-R intervals, abnormal or rapid heart rate, and reduced blood pressure (11).

AF also appears to be associated with the risk of developing Alzheimer’s disease. Numerous studies suggest that the occurrence of Alzheimer’s disease is related to hypoperfusion, inflammation, oxidative stress, and endothelial dysfunction. In addition, several circulating biomarkers of oxidative stress, inflammation, and endothelial dysfunction have been demonstrated to be elevated in AF (12). As these factors are also associated with dementia, it seems conceivable that AF, through these pathways, may be related to cognitive decline and dementia.

Several studies are currently investigating primary or secondary effects of various therapies, including anticoagulation and interventions, on cognitive function in patients with AF (13).

Knowing the close association between AF and dementia, further studies on diagnosis and therapy are of extraordinary clinical importance. Pulmonary vein isolation is the most effective therapy for paroxysmal and persistent AF. According to positive studies in recent years, pulmonary vein isolation is also expected to prevent deterioration of neurocognitive function (1). This question is addressed by Zwimpfer et al. in their prospective, multicenter SwissAtrial Fibrillation Cohort study (Swiss-AF) in which patients who underwent pulmonary vein isolation were included and compared with patients who were treated conservatively. Neurocognitive analysis included several tests, including the Montreal Cognitive Assessment, and was performed at study inclusion and after one year. Notably, this study revealed no association between treatment by pulmonary vein isolation and neurocognitive function. The authors note that there have been studies in the past with contrary results. In addition, the number of patients with pulmonary vein isolation in the present study is low. Nonetheless, Zwimpfer et al. provide an important hint that influencing neurocognitive function with pulmonary vein isolation has to be questioned (Zwimpfer et al.).

Intervention studies that aim to investigate the benefit of pulmonary vein isolation, for example, have the fundamental problem that years and decades may pass between intervention and the onset of dementia. What is needed, therefore, are parameters of cognitive function that could measure subtle changes after just a few months or years. In this special issue, Lai et al. demonstrated that use of the telephone Montreal cognitive assessment in patients with AF is appropriate for screening mild cognitive impairment. The relatively simple telephone screening could be useful for further studies (Lai et al.).

**Figure 1:**
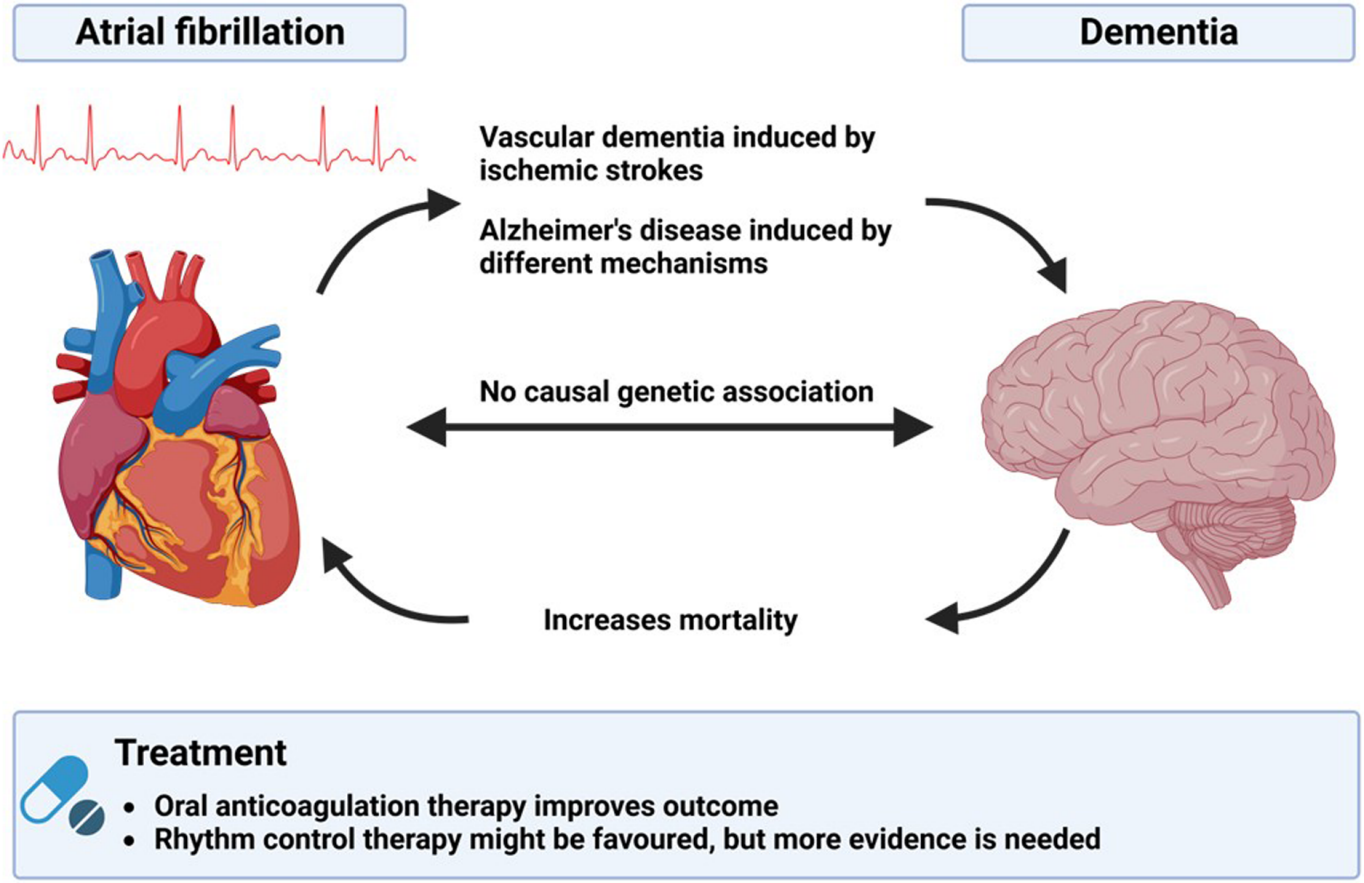
Illustration of the interactions between atrial fibrillation and demetia and possible therapeutic options.

Overall, this special issue of Frontiers in Cardiovascular Medicine provides an insight into the pathophysiology and epidemiology of AF and dementia. It also highlights the pitfalls and limitations of dementia prevention. Figure 1 illustrates the results of the discussed publications (Figure 1). As AF and dementia will increase in clinical importance in the future, further research is of utmost importance.
